# Correction: Influence of the primary and secondary coordination spheres on nitric oxide adsorption and reactivity in cobalt(ii)–triazolate frameworks

**DOI:** 10.1039/d1sc90237g

**Published:** 2021-11-12

**Authors:** Julia Oktawiec, Henry Z. H. Jiang, Ari B. Turkiewicz, Jeffrey R. Long

**Affiliations:** Department of Chemistry, University of California Berkeley California 94720 USA jrlong@berkeley.edu; Department of Chemical and Biomolecular Engineering, University of California Berkeley California 94720 USA; Materials Sciences Division, Lawrence Berkeley National Laboratory Berkeley California 94720 USA

## Abstract

Correction for ‘Influence of the primary and secondary coordination spheres on nitric oxide adsorption and reactivity in cobalt(ii)–triazolate frameworks’ by Julia Oktawiec *et al.*, *Chem. Sci.*, 2021, DOI: 10.1039/d1sc03994f.

The authors regret that incorrect details were given for ref. 35, 37 and 59 in the original article. The correct versions of ref. 35, 37 and 59 are given below as ref. 1, 2 and 3, respectively.

The Royal Society of Chemistry apologises for these errors and any consequent inconvenience to authors and readers.

## Supplementary Material
